# “We’re getting older and things are changing”: Qualitatively exploring older adults’ everyday experiences in relation to early changes in mobility

**DOI:** 10.1371/journal.pone.0345782

**Published:** 2026-05-13

**Authors:** Olivia Chivers, Lynn Rutledge, Marla Beauchamp, Brenda Vrkljan, Evelyne Durocher

**Affiliations:** 1 Faculty of Health Sciences, School of Rehabilitation Sciences, McMaster University, Hamilton, Ontario, Canada; 2 Rehabilitation Sciences Institute, University of Toronto, Toronto, Ontario, Canada; Instituto Nacional de Geriatria, MEXICO

## Abstract

Mobility can require skills from ambulating to driving and using public transportation. The onset of mobility impairments has been linked to reductions in physical function, social participation, health status and quality of life. We qualitatively explore how early changes in mobility are experienced by older persons and how such changes shape performance and engagement in everyday activities in later life. We conducted semi-structured interviews with 25 community-dwelling older persons and through thematic analysis identified four themes in the data. Participants described perceptions that everyday activities require more energy, a need for increased awareness and mitigation of risks when doing activities, a “use it or lose it” approach to maintaining mobility and comparisons between themselves and others. The results suggest that addressing expectations of aging, promoting engagement in meaningful activities, particularly those that enhance mobility, and increasing awareness of early changes in mobility are critical to maintaining engagement in later life.

## Introduction

Mobility is an important aspect of daily life, yet complexities of mobility can be underappreciated. Defining mobility can be challenging in light of variation in how human beings move within their homes, communities and beyond. Movement for mobility frequently involves using our joints and muscles to complete actions such as getting in or out of a chair or ambulating, and may include the use of assistive devices, such as a wheelchair or cane. Transportation is also a predominant aspect of mobility; access to a car and other modes of sustainable and accessible transit allow people to get to places and go further afield. [1,2]. In their community-based work focused on community-dwelling older persons and how they remain independent, Stalvey and colleagues [3] describe mobility as “travel[ing] in, around, and outside the home as one conducts the business and social aspects of everyday life” (p. 461). This definition highlights an important consideration of human-context transactions in relation to mobility: we often move with a purpose.

For older persons, loss of mobility has been associated with lower life satisfaction and depression. [4] Additionally, physical changes, such as impaired balance and gait, in conjunction with the development of various medical conditions, all of which are more common in later life, can increase risks of falls, a leading cause of injury, hospitalization and death for those in this age group. [5,6] Early changes in mobility, sometimes called pre-clinical mobility limitations), reflect “subtle and often unconscious change[s] in the ways older people move” and are changes that could easily be missed [p. 1647,7]. Little is known about such changes, making them more difficult to detect, despite claims that identifying and addressing mobility changes as early as possible, and in particular in individuals “who are not currently reporting mobility difficulty but who may be at rks for more definitive functional change”, is critical to maintaining quality of life into later years [p. 1647, 7].

Factors demonstrated to be related to mobility changes in older persons have typically been measured using quantitative data. A focus on quantitative measures is evidenced in papers such as the recent scoping review of physical measures of mobility Kalu and colleagues’ [8] or the work of Bechtold, Stauder and Fieder [9] who examined older persons’ quality of life in relation to mobility status using rating scales. Few studies use qualitative methods to investigate the experiences and perceptions of older persons in relation to mobility changes, which may illuminate important considerations that cannot be easily identified or explored using quantitative methods. In her landmark paper, Tinetti [10] described mobility changes as resulting “from a complex set of interactions among intrinsic and extrinsic factors” (p. 126). Tinetti argued that mobility cannot be captured using a medical lens through which each factor is examined independently, rather, she argued that examining a combination of intrinsic and extrinsic factors in conjunction with an individual’s life experiences, both past and present, should be utilized to explore individuals’ mobility changes in later life.

In response to Tinetti’s call for more exploratory research in the area of mobility and aging, a body of literature qualitatively describing mobility change is emerging. This literature includes for example the metasynthesis of qualitative research by Goins and colleagues [11] exploring perceptions of mobility by older persons. Most qualitative studies investigating mobility change in older persons however have focused on people with limited mobility or disability; less consideration has been given to how these changes first occur when individuals may not necessarily recognize that such changes are occurring [7]. Qualitative examination of this stage can offer a more open exploration and to compel individuals to look deeper than what might be expected in order to broaden our understanding of what exactly might comprise such changes. This knowledge can then inform future more targeted research and the development of approaches to identify early mobility change that could otherwise risk being undetected and unreported. This knowledge can further inform how initial changes may be addressed, early on, to prevent mobility issues from developing or developing further [7]. In the current study, we take a qualitative approach to explore how early changes in mobility are experienced by older persons and how such changes shape their performance and engagement in everyday activities in later life.

## Methods

### Study context

The study was set in a mid-size urban centre in Ontario, Canada, in which approximately 18% of the population is aged 65 years and older. [12] The urban centre is served by multiple transit systems including a local bus service, an intercity transit system consisting of both bus and train routes, as well as a specialized transit service for disabled and aging clients. Surrounding the downtown core lay a variety of suburbs and neighbouring cities, which most study participants reported accessing by personal vehicles. Due to the multiple transit options, mix of urban and suburban lifestyles, and proportion of older persons in line with the national average [12], this city provided an optimal location for investigating perspectives of older persons on changes in their mobility.

### Participants and recruitment

Older persons living in the community were recruited between January and November 2021 through newsletters targeted to older persons and advertisements in local newspapers. Once enrolled, participants were asked to share study information with others in their personal network. Potential participants were asked to contact a member of the research team by telephone or email. In an initial phone call, the study purpose and process were explained and interested individuals were screened for eligibility. Individuals were eligible to participate in the study if they were 65 years of age or older, residing within the community, able to communicate in English and able to provide informed consent. Individuals living in long-term care or other institutional settings were excluded, as were those with mobility loss from a traumatic event (such as a stroke or accident) because such events would not likely enable the experience of early changes in mobility, which are the focus of this study.

### Data generation

Semi-structured interviews were conducted by telephone or Zoom (according to participant preference) between January and November of 2021. In-person contact was not permitted for this study given the context of the COVID-19 pandemic. As endorsed by the research ethics board approving the study (Hamilton Integrated Research Ethics Board, project #7594), informed consent was obtained, audio-recorded and witnessed by the researcher conducting the interviews at the beginning of each interview. Prior to their interview, each participant was asked to identify two photographs: one exemplifying mobility in their life in the past and one exemplifying mobility in their life today. They were invited to share this photograph by email or through the Zoom camera with the interviewer at the beginning of the interview. The request to identify photographs was intended to stimulate participants’ deepened reflection prior to the interview about mobility in their lives and cue memories of activities not present in daily life at the time of the interview due to reasons such as pandemic restrictions or mobility change. This approach was guided by methods of photo-elicitation, in which photos are used for “meaning sharing and meaning constructing between the participant and the researcher” [p.12, 13]. Sharing the photographs with the interviewer was intended to prompt participants to reflect about mobility throughout their lives prior to the interview and thus incite deeper conversations about mobility changes and the meaning of mobility in each participant’s life. In addition, during their interview, each participant was asked to stand from a sitting position and then to sit back down again, describing each part of this process. These movements also prompted further discussion about changes that might be experienced in relation to mobility. Interview questions included for example: Can you tell me about the activities you are doing in the photographs? What does/did [activity] mean to you? How is it doing this activity now? Can you tell me about how you get around your home/neighbourhood today? Have you noticed any changes in how you get around/in how you do your activities? What does mobility mean to you?

### Data management and analysis

Interviews were professionally transcribed; the transcripts were reviewed for accuracy by members of the research team prior to being uploaded to Quirkos software for data management. An initial codebook was created by two research team members based on the research questions. These team members then coded three transcripts each and met to refine the codebook, revising codes as necessary. Each transcript was then coded independently by two researchers using the refined codebook. Following the coding stage, the data was analyzed by one researcher in close collaboration with the research team using methods of thematic analysis as described by Braun and Clarke. [14]

## Results

Twenty-five participants from the Greater Hamilton area were recruited. All participants were older than 65 (mean age of 76.28). In terms of mobility, during the initial screen, 20 participants self-reported no change in mobility, 2 reported mild change, 3 reported moderate change, and 0 reported major change. Of the 25 participants, 15 identified as women and 10 identified as men. Please see Table 1 for participant information. Interviews lasted between 46 and 120 minutes in length (mean 68.04 minutes).

**Table 1:** Participant characteristics.

Participant ID	Gender	Age	Self-identified mobility change status
1	Man	82	Moderate
2	Woman	79	None
3	Woman	74	None
4	Woman	71	None
5	Woman	80	Minor
6	Woman	78	None
7	Man	75	None
8	Woman	75	None
9	Man	76	None
10	Woman	77	None
11	Woman	78	None
12	Woman	71	None
13	Woman	74	None
14	Woman	69	None
15	Woman	76	None
16	Man	70	None
17	Woman	80	Minor
18	Man	80	Moderate
19	Man	79	None
20	Man	80	None
21	Man	84	Moderate
22	Woman	66	None
23	Man	75	None
24	Woman	86	None
25	Man	72	None
	Men n = 10, Women n = 15	Mean: 76.28 years of age	None n = 20 Minor n = 2 Moderate n = 3

Interestingly, despite the high predominance of participants reporting no or mild changes in mobility (20 reporting no changes, 3 reporting moderate changes and 2 reporting mild changes), in their interviews, all participants reported changes in their experiences and perceptions of mobility, as well as changes related to mobility in how they approach their daily activities, thus indeed suggesting the experience of early changes. Such changes are explored below in the four major themes identified through the analysis: everything takes more energy now; increased awareness and mitigation of risks; “use it or lose it”; and evaluating mobility through comparisons to others.

### Everything takes more energy now

Across the narratives, participants described how they noticed subtle changes in their mobility across time. These changes included moving more slowly than before, taking more breaks, and becoming increasingly sedentary. Reports of feelings of fatigue and reduced stamina were also identified in the data.

Some participants suggested that aging was inevitably linked to slowing down and having less energy. Participant 3 commented: “*look at someone who is 10, 15 years older than me, [these people] are a little slower…I don’t know if those things can really be altered.*” For some participants, slowing down and limiting activities was linked to decreased energy levels. Participant 2 stated: “*certainly I don’t have the energy I had when I was younger. And as a result…maybe I can’t jog anymore*”; participant 7 stated that as an older person *“[one] probably couldn’t walk or hike as far as [one] did when [they] were young, and [one] gets fatigued more easily. So obviously that’s a given*”. The use of definitive language such as “*certainly*”, “*obviously*” and “*a given*” in the quotations above is suggestive of beliefs that slowing down and having less energy are inevitable as one ages.

Decreasing energy and slowing down were also identified in participants’ discussions of their daily activities and reports of taking more breaks. Participant 3 described previously spending long periods of time standing: “*I had a job in a bank, and I always stood up. I stood up all the time. When I was at home, I was still standing up. I couldn’t go sit down*.” Reflecting on their current mobility, they commented: “*I’ll cook in the kitchen for a while, and then I’ll come and sit down. Whereas before I maybe wouldn’t go and sit down*.” This participant noted changes through their increasing use of seated breaks. Participant 13 commented on their level of activity and corresponding mobility: “*normally I do physical activity in the morning - in the garden, on the farm, hiking - and then by the time 1:00 comes around, I’m thinking, ‘Ah, it’s time for a rest.*’”, For this participant, taking a rest had become part of their daily routine. Another participant described needing more breaks and even requesting an environmental adaptation in their community:

*I amble slowly with frequent rest stops. So in my hour walk during the day with the one dog, if I* *’* *m feeling good I don* *’* *t have any stops. But there [are] some days when I have to sit on the bench a couple of times. I have gotten them to install a bench just by the water in the park. And so I always stop there. (Participant 24)*

Some participants reported that initiating an activity required more energy. They noted this additional exertion when starting a walk or rising at the start of the day. This change was most reported in the context of transitioning between sitting or lying down and standing. Participant 16, for example, stated:

*When I first get up and I’m limping for the first hundred steps or so, they say, ‘What* *’* *s the matter with you? Are you getting old?’ Actually, I* *’* *m getting older. But this will be okay. Give me a hundred steps and I* *’* *ll be okay* *.*

This participant described their non-reciprocal gait as a physical manifestation of their decreased mobility. Similarly, Participant 20 used the term ‘pause’ to describe the moment when they feel difficulty but that this moment then passes: “*I’ll just pause when I get up, and then I’m fine.*” Overall, most participants discussed fatigue, slowing down, taking breaks and initiating activities, which they perceived as an expected part of aging.

### Increased awareness and mitigation of risk

Throughout the interviews, many participants reported being more aware of physical changes and potential risks of injury, particularly in relation to falls. They described being more aware and cautious now compared to when they were younger. Participant 21 described being more cautious and deliberate even prior movement:

I*f I’m going to move from point A to point B, I think about it first. How am I going to get there? Do I have something to lean on if I feel like I’m falling? Everything is carefully thought out. Whereas things used to be automatic, now it’s got to be carefully thought out.*

Some participants shared how their movements had once been unconscious or as Participant 21 said, “automatic”, compared to now being more deliberate and being “carefully thought out”, suggesting a heightened awareness of risks of falling as they age.

Some participants described consequences of falling in older age in a way that linked aging to inevitable decline and frailty. For example, participant 12 shared that they “*read a lot of studies... the trajectory after people fall is pretty nasty*”; participant 2 stated that they “*hear stories about older people slipping and falling, and [older persons are] more prone to breaking bones*”. Both participants imply an expectation that the consequences of falling become greater as one ages, an expectation that they report is informed by research and anecdotal evidence.

Heightened awareness of physical risk was reported to be informed by participants’ personal experiences of falls; increased awareness and more cautious behaviour were intended to decrease risks of falling. Participant 3 shared how their amplified awareness resulted from experiencing a fall and being injured: “*I’m aware of the sidewalks. Because two years ago, I broke my wrist.*” They further shared that “*looking down at the sidewalk... is an annoyance but I have to look down to make sure I am not going to trip and break my other arm.*” In this instance, cautious behaviour was directly motivated by wanting to avoid another fall. Personal experiences of falling thus shape participants perspectives of risk.

Many participants described stopping activities to avoid risk of injury. For example, participant 7 reported stopping skiing for fear of falling, while participant 13 no longer “*cycle[s] because [they are] afraid of falling off and shattering something.”* Some participants reported choosing to continue activities but avoided participating under conditions deemed riskier. Participant 8 for example reported continuing to walk in their neighbourhood but not going out “*at night when there was ice and snow*” as they “*didn’t want to slip. You can’t always see ice in the dark*.” Other modifications in activity contexts were reported by participant 18:

*I tend to walk on [main streets] rather than on side streets. Because if I go down [on side streets], nobody will see me... If I fall, there’s more chance of people walking or biking or riding on those [main] streets than there is on [side street]* *.*

For many participants, being more cautious meant adding and using safety equipment, such as stair railings, grab bars and anti-slip bathmats, in their everyday environments. When asked about climbing stairs in their home for example participant 11 said that they “*always hold on [to the railing] going down because [they are] trying to be intelligent about things... to be cautious*.” In this case, being “*intelligent*” and “*cautious*” imply mitigating risks of falling.

Increased awareness of risks and taking greater caution were extensively discussed in the interviews. These risks were suggested to be informed by experiences of falling by the participants and others, and expectations of aging.

### “Use it or lose it”

A theme identified across interviews related to the importance of maintaining mobility is what participant 10 described as “*use it or lose it*”. Multiple participants described a desire to maintain their current level of mobility through activity and exercise and a fear of losing mobility if they did not engage in such activity. While many accounts reflected perceptions of decline, a few of the participants suggested their mobility was improving due to higher levels of exercise than previously in their lives. When asked about what mobility meant to them, many participants emphasized as participant 12 said: “*freedom and independence*”. Participant 6 described mobility as being closely intertwined with their independent performance of activities of daily living:

*I* *’* *m able to take care of my home. I can clean my condo. I can do my own cooking. I can go and get my own groceries. I can drive my car. I can pay my own bills. I just feel that I can maintain the way I had been living* *.*

Mobility was thus described as enabling freedom and being able to complete daily activities independently, which participants described as being “*very, very important*” (participant 6).

Loss of mobility was described in negative terms; participants often described seeing the impact of such losses in their peers. For example, participant 23 described:

*We have several [neighbours] in their 80s and 90s. And the ones who are mobile, you notice. And the ones who aren’t, it’s pitiful. They’re sad. They can* *’* *t move… But the ones who are up there in their 80s and 90s that are still walking, driving their car, that* *’* *s fantastic* *.*

By using words like “*pitiful*” and “*sad*” to describe neighbours with limited mobility, and words like “*fantastic*” to describe others who are more mobile, participant 23 is suggesting that not only is being mobile very important, but that diminished mobility is bad and to be avoided. Participant 10 said, *“Other than the railing on the stairs, no [I don’t use safety equipment]. I would say I’m pretty normal.”* suggesting that reliance on mobility aids is abnormal and should be avoided.

Participants discussed low activity levels as leading to an undesired result of decreased mobility. Participant 12 for example suggested that one’s mobility levels “*will change [for the worse] if you don’t work on it*”. Participant 8 discussed not enjoying gym exercises but continuing with them as “*self-preservation to keep [their] knee from stiffening*”. Participant 10 stated that they had increased their exercise levels as they aged because “*exercise [and] walking will do nothing but improve your physical being as you get older.*” By describing changes that participants have made to their lives, it is suggested that exercise is viewed as an investment in maintaining mobility as one ages.

### Evaluating mobility through comparisons with others

As illustrated above, some participants used others’ mobility as a subjective gauge by which to evaluate their own mobility and corresponding activity levels. Participant 25 for example said that they “*do more than [their] neighbours*.” However, when describing their wife, stated, “*my wife is very active. She would like me to do more*”. By comparing their own mobility to that of others, this participant demonstrated their shifting evaluation of their own mobility. In some cases, comparisons to others shaped participants’ aspirations of maintaining mobility and levels of activity. Participant 13 stated:

*I look at my mother... I realized the more she didn* *’* *t [move], the more she couldn* *’* *t do it. And in the end, the last five years of her life, she was in a wheelchair. It wasn* *’* *t anything wrong with her except that she had stopped doing everything and I don* *’* *t ever want to be like that. Then I look at my husband* *’* *s father who was driving his car and curling right up until the very end. He was 103 when he fell and hit his head and had a stroke with a head injury. He kept going all the time... Seeing those two role models, I intend to keep going all the time* *.*

Comparisons to others motivated participants to emulate particular behaviours and promoted a sense of competition, even with much younger generations. Participant 4 described pushing themselves to complete exercises in comparison to the performance of others indicating “*if I’m working in these classes, and there’s somebody who’s 40 or 50 and they can do it, damn, I’m doing them. That keeps me moving*.” By comparing their mobility to younger peers and to their own expectations of aging, participants framed perceptions of their own mobility, using such comparisons to shape their goals and influence shifts in their mobility levels.

This analysis suggests that as one ages they have less energy and need to be more diligent and aware of risks of falling; if one does not use what they have, they lose their abilities, however comparison with others can be a powerful motivator to maintain mobility. Participants reported a wide variety of factors and contexts that shape their perceptions of their own mobility including feelings and values, previous experiences, observations of others and the environmental context. Early changes in mobility also varied widely, with the most common changes being slowing down, taking more breaks, and discontinuing activities deemed risky.

## Discussion

In this study, we explored how early changes in mobility are reflected in the perceptions of older persons as they engage in their everyday activities, despite most participants not identifying any changes in their mobility. The analysis presented suggests that changes in intrinsic factors – such as having more pain, anxiety or fear when doing an activity, or taking breaks – as well as interactions with extrinsic aspects in one’s context or environment – such as increased use of handrails and benches- were some of the subtle changes noted by participants as early indicators that their mobility was changing or impaired. These factors align with the work of Tinetti (1986) who described mobility change to be impacted and reflected not only by changing abilities or physical decline but also an array of factors such as anxiety, social context, and environmental features such as uneven ground and stairs. [10] In the present study, participants discussed a broad and far-ranging array of experiences and perceptions of mobility changes, thereby supporting Tinetti’s emphasis that mobility changes are heavily dependent on a complex and nuanced web of intrinsic and extrinsic contexts.

In the narratives there was much variation in what was described as changes in and affecting participants’ mobility. Major themes identified in this study are in alignment with early changes in mobility that have been described in the literature. [15,16] Slowing down, decreasing and avoidance of certain activities, and taking breaks are all examples that have been previously linked with mobility impairment. [15,17,18] Artuad and colleagues [17] go one step further by identifying slow walking speed as not only a potential predictor of mobility change and disability in later life but also of further and significant declines in mobility to chronic conditions. While many of the current study participants similarly described slowing down as an early mobility change, in the majority of discussions slowing down was attributed by participants to be a function of aging and decreasing energy levels, rather than as explicitly linked to disease. These findings align with those of a longitudinal study conducted by Knoop and colleagues [19] in which the authors reported that self-perceived fatigue predicted slower gait and lesser function when performing activities of daily living. The current study builds on these ideas by showing a picture of why older persons may slow down and provides important insights into how some older persons are responding to fatigue, a key factor that may be considered when addressing declines in gait speed for example.

Although prior to the interviews most participants in this study indicated they had not experienced physical changes, most described a fear of falling – which was frequently linked to an experience of a fall – and reported heightened vigilance of risks in the environment. These findings are in alignment with those of Lee and Tak [20] and MacKay and colleagues. [21] Lee and Tak [20] noted an unease related to a sense of vulnerability perceived to be linked to aging; such findings are echoed in this study when participants considered decreased mobility to be an inevitable result of aging. A fear of falling may present a psychological barrier where people change their participation in activities of daily living or avoid such activities altogether. Fear of falling was an often-reported reason for activity discontinuation. In their study, Lee and Tak [20] also describe activity curtailment as a consequence of fear of falling, which is supported by several anecdotes shared by study participants. These findings are also consistent with a 2021 longitudinal study conducted by Liu and colleagues [22], who suggest that both fear of falling and fall experiences limited daily activities. The experience of a fall has a lasting impact on participants’ choices of activities and environments, which can further limit their mobility and health both in the short and long term.

An interesting consideration is that across the interviews there was little reflection or discussion of public transportation or transit use. Participants in this study tended to consider mobility as their own movements and abilities, particularly walking, but also as their use of a personal vehicle. Such views may reflect the transportation patterns of most older Canadians, who predominantly travel in personal automobiles as either drivers or passengers. [23] We also acknowledge however that the interviews were conducted during the COVID-19 pandemic, which may also have shaped reflections of the use of public transportation.

A closer examination of how participants perceived their own changes in mobility and those of others is suggestive that these were often viewed with an ageist lens. Ageism is defined as stereotyping and discrimination on the basis of age, particularly affecting older persons. [24–26] Ageism can be expressed towards others through institutional and interpersonal means but can also be also internalized by individuals. [26] Expressions of ageism were noted in participant anecdotes such as in expectations that people older than them would move slower or when the use of mobility aids was discussed using negative or ableist terms. While reflections of ageism in the narratives were not reported to directly affect mobility change, internalized ageism may shape perceptions of mobility change during the aging process and may thus influence further changes in mobility. Bailey and colleagues [27] for example suggested that ageism is a contributor to delays in the installation of mobility aids in the home. Links between fear of physical deterioration, motivation to improve mobility and corresponding increases in activity have also been identified in the work of Maula and colleagues [28] and of Rasinaho and colleagues, [29] and are reflected in the current study’s theme of *use it or lose it*. In their mixed theoretical and empirical study however Zingmark, Ankre, and Wall-Reinius [30] suggest that older persons may withdraw from activities based on their perception of their own (older) age, regardless of their level of mobility. Expectations and perceptions of aging may thus contribute to cessation of some activities. Internalized ageism may also cause older persons to conform to their society’s age stereotypes, thereby limiting their own sense of individualized occupational identities and possibilities, and furthermore resulting decisions about the occupations in which they engage as they age. [26] Given that expectations of aging were identified in the analysis and have been shown in previous research [28–30] to shape activity and exercise participation, addressing internalized ageism is likely important in maintaining mobility and engagement in daily activities.

### Strengths and limitations

Strengths of the study include the approach to the interview, which included asking the participants to share personal pictures representing their own mobility in both their past and present activities as well as asking participants to stand and sit during the interview. The exercise of finding photos prompted participants to consider mobility in their daily lives and how their mobility may have changed. Asking the participants to stand and sit prompted specific consideration of their immediate experience of these elements of mobility. Such approaches promoted more depth of examination by the participants of their experiences of mobility. Another strength of the study is the variety of living situations of participants (e.g., living alone or with a partner, in urban or semi-rural settings) resembles the variation in how older Canadians live. This diversity may allow for a better picture of the range of mobility patterns and contributors to mobility change of older persons, and in turn may increase the external validity of the study.

A limitation of the study is that the participants were recruited from the community on a volunteer basis. There may be a self-selection bias in that individuals who are more concerned with their mobility and thus keeping more active being more likely to join, including both those who are more active and mobile, and those who experience more significant mobility difficulties, limiting the transferability of the research findings. A second limitation is related to the timing of the research, being conducted during the pandemic during a time when in-person interviews were not possible. Having telephone or online interviews as well as pandemic restrictions in place may have shaped participants’ reflections and ability to consider their mobility a few months prior at a time when pandemic restrictions were not in place. We attempted to overcome this limitation through the use of a photo to prompt reflection, which while a reasonable facsimile, may still have limited accurate reflection for participants.

## Conclusion

Variations in mobility changes and perceptions of these changes by participants in this study highlight the hetereogeneity of experiences of aging and corresponding mobility as a process. Participant perceptions of mobility were shaped by changes noted in their own abilities as well as those of others, and included physical experiences such as having a slower gait or relying on external supports. Perceptions also reflected an intricate network of internal feelings such as fear of injury or decline, values such as independence, and predetermined expectations of the aging process. Our results suggest that addressing ageism, promoting engagement in meaningful activities including those that support and enhance mobility, and increasing awareness of early changes in such activities may be the first step to introducing strategies that decrease risk and support meaningful engagement. Such recommendations could be explicitly implemented clinically through the use of workplace initiatives championing the elimination of ageism in perspectives; such initiatives would be a start but would also require investment at multiple levels given how ageism prevails at all levels of society [26,31]. Efforts to combat ageism are underway from multiple organizations and include specific curriculum for students of all ages as well as in health and social care provider and policy programs [for examples see 32–36]. Attunement in individuals of all ages including health and social care professionals to early changes in mobility could be enhanced again by workplace initiatives, education programs and public health campaigns. As the life expectancy increases, so should investment in the wellbeing of older persons, including supporting their mobility. Future research should build on this work to further refine our understanding of what early changes in mobility might entail and include a focus on exploring what older persons might find useful in helping them to manage changes in mobility earlier in the lifespan. The importance of mobility to older persons was underscored in this study. There is a need for further development of approaches to mitigate early declines in mobility to promote older persons’ engagement and satisfaction in their daily lives as they navigate the aging process.
